# Role of Enhanced Recovery After Surgery (ERAS) Protocols in Arthroplasty: A Systematic Review of RCTs and Prospective Studies (2018-2024)

**DOI:** 10.7759/cureus.90560

**Published:** 2025-08-20

**Authors:** Rakan M Aldossari, Ashaq Alqahtani, Mohammad S Alqahtani, Salah Alzahrani, Abdulrahman A Alghamdi, Mohammed A Saddah, Hussam Darraj

**Affiliations:** 1 Orthopedic Surgery, Aseer Central Hospital, Abha, SAU; 2 Arthroplasty and Reconstruction Surgery, Abha Hospital, Abha, SAU; 3 Orthopedics, Aseer Central Hospital, Abha, SAU

**Keywords:** enhanced recovery after surgery (eras), rehabilitation after surgery, systematic review, total hip arthroplasty (tha), total knee arthroplasty (tka)

## Abstract

Enhanced Recovery After Surgery (ERAS) protocols constitute evidence-based intervention pathways prepared to decrease surgical stress, maintain physiological function, and improve recovery. This study aimed to systematically review the ERAS effect after primary total hip arthroplasty (THA) or total knee arthroplasty (TKA) on patients' clinical outcomes. This systematic review was developed following the Preferred Reporting Items for Systematic Reviews and Meta-Analyses (PRISMA) guidelines. Online search was done through MEDLINE, PubMed, Cochrane Central Register of Controlled Trials (CENTRAL), Scopus, and Ovid. The search included the time from 2018 to 2024. The inclusion criteria included prospective nonrandomized cohort studies and RCTs that compared adult patients undergoing primary THA or TKA with ERAS or traditional (control) protocols. Studies that were conducted among patients with trauma and malignancies were excluded. The primary outcome was the length of hospital stay. The secondary outcomes included operation time, readmission rate, and intraoperative blood losses. Of the 642 records identified, six studies met the inclusion criteria. In total, 7206 individuals were analyzed, of whom 2148 underwent ERAS (intervention), and 5058 underwent traditional care either as a control or a pre-intervention group. Narrative synthesis was performed, and the ERAS interventions were divided into preoperative, intraoperative, and postoperative interventions. The systematic review showed that ERAS protocols significantly reduced hospital length of stay. There was no significant effect on readmission rates. However, intraoperative blood losses significantly decreased with the application of ERAS protocols. Regarding THA and TKA, ERAS protocols significantly decreased the length of hospitalization. The effect of readmission was comparable between the intervention and control groups. The impact of ERAS on operation duration was inconclusive. Further research is needed to assess and analyze the definitive impact of each component of the ERAS protocol on total joint arthroplasty.

## Introduction and background

Many patients receive relief from the severest of joint conditions through arthroplasty, especially hip and knee replacements. However, postoperative care has too often kept patients hospitalized, prolonging their stays in hospital beds for far too long, and it also delayed patients from bending or straightening their hip or knee and increased adverse events as well as complications [1]. Patients undergoing primary hip and knee surgeries sometimes require readmission within 30 days of surgery [2]. Therefore, Enhanced Recovery After Surgery (ERAS) protocols have been developed to overcome such challenges and optimize patient outcomes and well-being [3].

ERAS protocols constitute evidence-based perioperative care pathways prepared to decrease surgical stress, maintain physiological function, and improve recovery. It was shown to reduce hospital stays by 30% to 50% [4]. Recent research has demonstrated the effectiveness of ERAS protocols in enhancing postoperative recovery. To elaborate, a systematic review and meta-analysis conducted by Zhu et al. shed light on the significant improvements in recovery parameters for patients undergoing hip and knee arthroplasty under ERAS protocols compared to traditional care pathways [5]. At the same time, Di Martino et al. focused on the benefits of fast-track protocols and early recovery and management in total hip arthroplasty (THA), which aligns with the ERAS protocol concepts [6].

In addition, positive outcomes in ERAS implementation and thromboprophylaxis following arthroplasty were reported and evident by Weng et al. in China [7]. Perioperative pain management is considered a critical component of ERAS, as de Ladoucette's review on postoperative pain management after total knee arthroplasty (TKA) further underscored and clarified [8,9].

Moreover, ERAS protocol implementation has been linked to clinical benefits and systemic improvements in healthcare delivery. For instance, Zhao et al. conducted a retrospective study demonstrating the positive impact of ERAS on patient outcomes and hospital efficiency in total joint arthroplasty (TJA) [10]. Additionally, the protocol for a systematic review by Rele et al. aimed to provide detailed insights and guidelines into the impacts of ERAS on TJA. Researchers found that this protocol could provide greater insight into the risks and benefits of reducing length of stay (LOS) for TJA. In addition, the research helped policymakers better understand whether reductions in LOS through ERAS pathways are occurring as an indicator of the patient and the health system [11]. In addition, a study by Vanni et al. developed ERAS in a high-volume orthopedic hospital, focusing on significant improvements in healthcare efficiency and patient satisfaction [12].

Furthermore, Morrell et al. concluded the experts' opinions and perspectives regarding ERAS, discussing its multidisciplinary approach and the resultant improved patient outcome. ERAS protocols for arthroplasty include preoperative patient optimization and intraoperative and postoperative interventions [13]. A study by Nag et al. emphasized the significance of a multidisciplinary approach in using ERAS for TKA, which resulted in better patient outcomes and reduced adverse events and complication rates [14].

To our knowledge, the ERAS protocol has proved its efficacy among groups undergoing TKA. However, there is a demand for a new, updated review of the recently published data regarding this protocol. Therefore, this systematic review evaluates recently published RCTs and prospective studies from 2018 to 2024. It aims to assess the ERAS protocols among hip and knee arthroplasty patients, focusing on their operation time, length of hospital stays, and readmission rates.

## Review

Methods

The study was conducted according to the principles of the Cochrane Handbook for Systematic Reviews of Interventions and reported following the Preferred Reporting Items for Systematic Reviews and Meta-Analyses (PRISMA) 2020 guidelines [1].

Eligibility Criteria

We included only RCTs and prospective studies published in English between 2018 and June 2024. Studies included adult male and female patients undergoing knee or hip arthroplasty.

Intervention

Direct comparisons were made between patients who underwent ERAS protocol interventions and those who received traditional therapy, standard-of-care protocol, were part of control groups, or were treated before the implementation of ERAS protocols.

Exclusion Criteria

The excluded studies were [1] studies with no full-access link; [2] studies with inappropriate objectives/outcomes; [3] studies conducted among patients with trauma, tumors, or malignancies; [4] study types such as case reports, letters, retrospective studies, review articles, systematic review articles, and studies without a control group; and [5] duplicate studies found in multiple databases or sources.

Search Strategy

An online search was carried out using five databases: MEDLINE, PubMed, Cochrane Central Register of Controlled Trials (CENTRAL), Scopus, and Ovid. We did not use any search filters, and the search included the time from 1st January 2018 to 1st July 2024. The terms used for searching included ("Enhanced Recovery After Surgery") AND (Arthroplasty).

Selection of Studies

Two independent reviewers conducted the online search processes, screening the titles and abstracts and revising the full text of relevant articles. Any disagreements were resolved by consensus.

Data Extraction

We extracted the following data: (a) study design, period, population size, and characteristics; (b) participants' age, sex, BMI, follow-up duration, and ERAS protocol used; and (c) outcomes including operation time for intervention and control groups, hospital LOS, and readmission rates.

Measured Outcomes

The primary outcome was the length of hospital stay. Secondary outcomes included operation-related clinical outcomes such as operation time, readmission, and intraoperative blood loss.

Assessment of the Risk of Bias in the Included Studies

We assessed the risk of bias (ROB) using the ROB2 tool for RCTs [15] and ROBINS-I for non-RCTs [16]. The ROB2 tool comprises five domains: randomization, deviations from the assigned treatment, missing data, measurement of the outcome, and selective reporting of the outcomes and results. ROBINS-I includes additional confounding and participant selection domains. Moreover, the overall ROB is assessed by selecting the highest level of ROB out of the five domains. The ROB figures were made using the Robvis visualization tool [17].

Results

**Figure 1:**
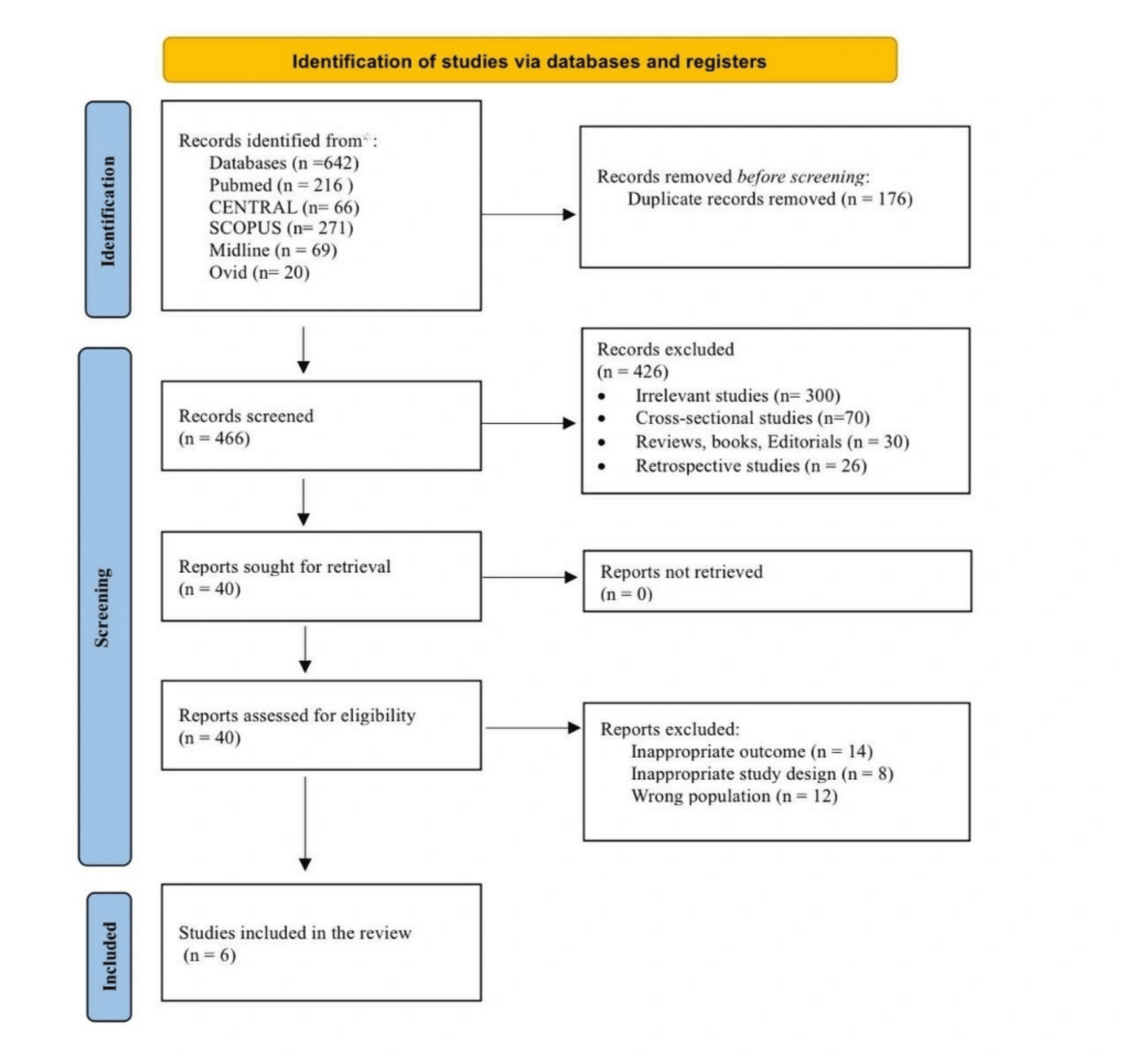
PRISMA flow chart for literature search and study selection PRISMA, Preferred Reporting Items for Systematic Reviews and Meta-Analyses; CENTRAL, Cochrane Central Register of Controlled Trials

The search strategy yielded 642 records, of which 176 were duplicates. The remaining 466 records underwent screening of their titles and abstracts, and 426 were excluded. The full texts of the remaining 40 records were retrieved. Out of these records, 36 articles were excluded as follows: studies with an inappropriate study design (n=8), inappropriate outcome (n=14), and a wrong population (n=12). Finally, six studies were eligible for inclusion in the present systematic review (Figure 1) [18-23].

The Basic Characteristics of the Included Studies

A summary of the characteristics of the selected studies is elaborated in Table 1. Studies were conducted across India, China, Spain, the Netherlands, and Australia and were published from 2018 to 2022. Only one study was multicenter [20]. Four studies [18,20,21,23] were prospective nonrandomized, and two [19,22] were RCTs. Sample sizes ranged from 49 to 6146 participants, with a mean of 1201 and a median of 239 participants. The surgical approach was not reported in one study, and the five included studies reported medial parapatellar, midvastus, and subvastus approaches for TKA and anterior approach for THA. The length of follow-up ranged from one month to five years, with a mean of 17.5 months and a median duration of nine months. Our primary interest, the length of hospital stay, was available for most studies.

**Table 1:** Characteristics of the included studies *Preintervention **Postintervention BMI, body mass index; TKA, total knee arthroplasty; THA, total hip arthroplasty; ERAS, Enhanced Recovery After Surgery; SD, standard deviation; FP, fast track protocol; RP, regular joint care protocol

Study ID	Azam MQ et al., 2022, India [18]	Wei B et al., 2021, China [19]	Ripolles-Melchor J et al., 2020, Spain [20]	Jiang HH et al., 2019, China [21]	Fransen BL et al., 2018, Netherlands [22]	Tan NL et al., 2018, Australia [23]
Study aim	To evaluate the clinical outcome of fast-track TKA using ER protocol	To investigate the impact of ERAS protocols on the clinical effect of TKA	To assess the association of ERAS protocols with complications in THA and TKA patients	To explore the safety and efficacy of the ERAS program for elderly TKA patients	To compare FP with a regular joint care protocol (RP)	To determine whether adding 'non-surgical' components to pre-existing 'surgical' components would improve recovery after THA
Study design and duration	Prospective control study from January 2018 to June 2019	RCT from October 2018 to June 2019	Multicenter prospective cohort from October 22 to December 22, 2018	A prospective controlled study was conducted from January 2014 to June 2016	A non-blinded randomized controlled clinical pilot study from May 2011 to June 2017	Prospective, before-and-after interventional study from January 2015 to August 2016
Sample size	465	69	6146	247	49	230
Intervention	275	35	1592	106	25	115**
Control	190	34	4554	141	24	115*
Age (intervention), years, mean±SD	65.8±9.2	65.77±4.51	69.67±9.65	74.2±6.3	64±9	64.6±10.44**
Age (control), years, Mean±SD	66.1±10.1	65.62±5.06	70.33±8.90	75.4±5.9	61±7	63.9±10.27*
Surgical site	TKA	TKA	THA or TKA	TKA	TKA	THA
Female (intervention), N (%)	198 (72%)	28 (80%)	930 (58.41%)	58 (54.7%)	14 (56%)	78 (67.8%)**
Female (control), N (%)	125 (65.7%)	27 (79.4%)	2650 (58.19%)	83 (58.8%)	15 (63%)	75 (65.2%)*
Male (intervention), N (%)	77 (28%)	7 (20%)	662 (41.6%)	48 (45.28)	11 (44%)	37 (32.2%)**
Male (control), N (%)	65 (34.2%)	7 (20.5%)	1904 (41.8%)	58 (41.1%)	9 (37%)	40 (34.8%)
BMI (intervention) kg/m^2^	24.6±4.5	26.66±1.19	29.53±4.82	32.1±5.1	28.7±3.5	27.49±5.5**
BMI (control), kg/m^2^	25.1±3.9	26.91±1.22	29.53±4.75	31.4±4.5	30.0±4.1	28.89±5.96*
Follow-up duration	24 weeks (6 months)	48 weeks (12 months)	4 weeks (1 month)	96 weeks (2 years)	240 weeks (5 years)	6 weeks
Surgical approach	Midvastus approach	Midvastus approach	NA	Medial parapatellar approach	A sub-vastus approach	Anterior surgical approach

In total, 7206 individuals were analyzed, of whom 2148 underwent ERAS (intervention), and 5058 underwent traditional care either as a control or a pre-intervention group. The intervention groups' mean age (SD) ranged from 64±9 to 74.2±6.3 years. Sex proportions ranged from 54.7% to 80% female for the intervention group and 58.19% to 79.4% for the control group. The mean BMI ranged from 24.6±4.5 to 32.1±5.1 kg/m² for the intervention group and 25.1±3.9 to 31.4±4.5 kg/m² for the control group.

Table 2 reports the different types of ERAS interventions that were commonly used in the included studies. We categorized ERAS interventions into preoperative, intraoperative, and postoperative interventions.

**Table 2:** ERAS protocol interventions ERAS, Enhanced Recovery After Surgery; LIA, local infiltration analgesia; PONV, postoperative nausea and vomiting

ERAS intervention	Azam MQ et al., 2022 [18]	Wei B et al., 2021 [19]	Ripolles-Melchor J et al., 2020 [20]		Jiang HH et al., 2019 [21]		Fransen BL et al., 2018 [22]		Tan NL et al., 2018 [23]
Preoperative interventions									
Joint function exercise					X				
Lung function exercise					X				
Oral multimodal analgesia	X				X				
Clear oral fluids up					X				
Carbohydrate loading	X	X	X		X				
Preoperative optimization and education	X		X						
Preoperative fasting			X						
(-) Preoperative duration of fasting									X
Thromboprophylaxis			X						
Blood management			X		X				
Antibiotic prophylaxis			X						
Intraoperative interventions									
Anesthesia									
Regional spinal anesthesia			X		X				
General anesthesia		X					X		
Increase in spinal anesthesia									X
LIA intra-operatively							X		
Short-acting opiates							X		
Intravenous dexamethasone					X				
Use of tranexamic acid	X	X	X		X				X
Goal-directed fluid therapy (fluid management)			X						
Temperature management			X		X				X
Omission of drains and catheters	X								
Interventions to PONV	X		X						X
Postoperative interventions									
Postoperative analgesia		X		X					
Multimodal oral analgesia					X			X	
Early mobilization		X		X	X			X	
Initiation of oral nutrition and mobilization	X			X					
Postoperative physiotherapy assessment								X	
Use of ice packs						X			
Predefined criteria for discharge from the hospital	X							X	
Postoperative glycemic control				X					

Outcome Measures

Five studies [18-22] showed a significant decrease in the length of hospital stay when comparing ERAS with control groups (P=0.0001, P<0.001, P<0.001, P 0.001, P=0.036), respectively (Table 3). However, the study conducted by Tan et al. [23] showed no statistical significance between the two groups. Researchers reported a mean length of hospital stay of 5.94±5.21 days among patients undergoing the traditional approach and 5.02±2.46 days among patients undergoing ERAS, which showed no significance.

**Table 3:** Outcome measures NA, not available; IQR, interquartile range; LOS, length of stay

Outcomes	Azam MQ et al., 2022 [18]	Wei B et al., 2021 [19]	Ripolles-Melchor J et al., 2020 [20]	Jiang HH et al., 2019 [21]	Fransen BL et al., 2018 [22]	Tan NL et al., 2018 [23]
Operation time for intervention (minutes), mean (SD)	95.3±9.2	84.66±3.38	84.00±28.94	72.4±13.5	102.1±20.4	65.3±28.18
Operation time for control (minutes), mean (SD)	97.1±9.8	83.85±4.14	93.33±29.66	73.5±15.6	76.5±15.3	67.2±22.77
P-value	0.04	0.379	NA	0.562	<0.001	0.274
Hospital LOS (days) intervention, mean±SD	3.9±2.1	3.11±0.32	4±1.48	9.6±1.6	3.7±1.8	5.02±2.46
Hospital LOS control (days), mean±SD	7.5±3.2	7.06±0.60	5±1.48	11.3±1.9	4.7±1.3	5.94±5.21
P-value	0.0001	<0.001	<0.001	<0.001	0.036	0.212
Mortality (%)	0	NA	NA	Intervention: 1 (094%); control: 3 (2.1%)	0	NA
Readmission intervention (%)	2 (0.007%)	NA	40 (2.5%)	NA	NA	8 (6.96%)
Readmission control (%)	1 (0.005%)	NA	78 (1.71%)	NA	NA	5 (4.35%)
P-value	0.9	NA	0.051	NA	NA	NA
Interoperative blood loss, intervention, mean ±SD	NA	185.20±9.70	216.67±185.38	123.7±26.8	261.0±200.8	291.67±244.00
Intraoperative blood loss, control, mean ±SD	NA	134.56±13.51	233.33±222.59	146.4±30.2	45.8±127.6)	283.33±225.23
P-value	NA	P<0.001	NA	P<0.001	P<0.001	>0.999

The surgical operation time was reported among all included studies, where it was found that the duration was statistically higher among the ERAS group compared with the traditional care (control group) in two studies (P=0.04, P<0.001) [18,22]. Despite that, the duration of surgery between the ERAS group and the control groups in Wei B et al. [19] (2021), Ripolles-Melchor J et al. [20], Jiang HH et al. [21], and Tan NL et al. [23] showed no statistical significance.

Individual intraoperative blood losses differed significantly between the ERAS and control groups among the three studies (P for the three studies was lower than 0.001) [19,22,23]. While the blood loss was reduced in the study by Ripolles-Melchor J et al., the significance was not reported [20]. The ERAS effect on hospital readmission rates was assessed in three studies [18,20,23]. No significant difference was found despite lower readmission rates in the ERAS groups (Table 3).

Risk-of-Bias Assessment

**Figure 2:**
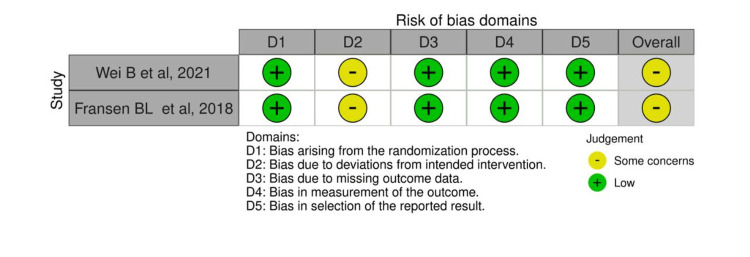
ROB traffic light plot for RCTs using the ROB-2 tool The evaluation approach is based on previously published tools and criteria [19,22]. ROB, risk of bias

**Figure 3:**
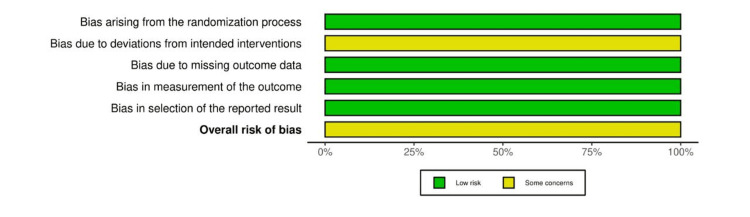
Summary of our ROB assessment for RCTs using the ROB-2 tool ROB, risk of bias

**Figure 4:**
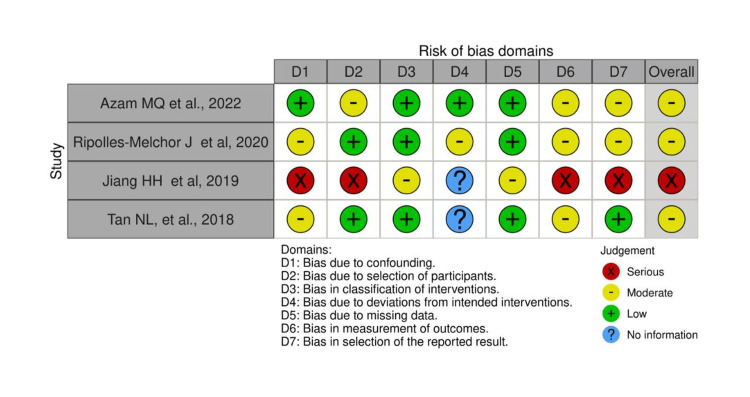
ROB traffic light plot for the prospective studies using the ROBINS-1 tool The evaluation approach is based on previously published tools and criteria [18,20,21,23]. ROB, risk of bias

**Figure 5:**
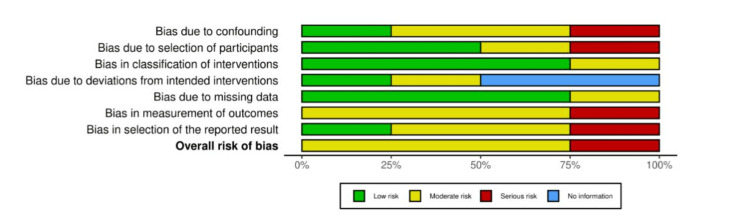
Summary of our ROB assessment for prospective studies using the ROBINS-I tool ROB, risk of bias

Five studies showed a moderate ROB, while one showed a high ROB due to bias caused by confounding factors, selection of participants, measurement of outcome, and selective reporting of the results [21]. Among the prospective studies, limited control for confounding bias, measurement, and reporting of outcomes biases were found to be common. Among the RCTs, deviation from the intended interventions due to blinding limitations was evident among the two trials [19,22]. In addition, other sources of bias were found in some studies. For instance, the statistical significance difference was biased due to the small difference between the comparable groups [18]. Furthermore, the small sample size might have introduced some bias in one study [19]. The results of methodological ROB for the included studies are demonstrated in Figures 2-5.

Discussion

ERAS protocol practices include multiple preoperative, intraoperative, and postoperative interventions to enhance patients' clinical outcomes and reduce mortality, morbidity, and complications. Such interventions have recently been discussed and published as a consensus statement by the ERAS Society guidelines for TKR and THR surgery. The practices include adjusting patients' educational conditions and anesthetic requirements, using a multimodal analgesic approach, and encouraging early mobilization. The outcome of the recent consensus showed that the protocol should be further evaluated and assessed to confirm its efficacy among various cases [24]. Another review concluded that implementing an ERAS program in emergency hip fracture repair successfully changed the healthcare processes and was associated with significant improvement in patients' clinical outcomes [25].

Our findings reveal that ERAS may significantly decrease the length of hospital stay among the majority of the included studies when comparing the ERAS group with the traditional treatment or control groups [18-22]. These findings offer solid proof for using ERAS interventions to safely shorten hospital stays after primary TJA. However, since most studies applied different perioperative and intraoperative ERAS interventions, deciding which ERAS components have the highest effect on the results and whether this impact can be sustained over larger patient groups and longer follow-up duration is challenging.

Previous systematic reviews assessed the ERAS protocol, comparing the ERAS treatment to control groups among knee and hip replacement surgery patients. The results revealed significant effects on clinical outcomes and complications [26-28]. However, our systematic review is the first that depends on recently updated RCTs and prospectively collected data, which adds more reliable evidence for the relation between ERAS and decreased length of hospital stay.

Concerning operation time, Azam MQ et al., 2022 [18], integrated preoperative optimization, oral multimodal analgesia, and predefined discharge criteria, while Fransen BL et al., 2018 [22], focused on blood management, regional anesthesia, and postoperative pain management using ice packs and multimodal oral analgesia. These components have contributed to enhancing surgical recovery. Furthermore, the process of positioning the patient and ensuring proper placement of the anesthesia can be attributed to prolonging the duration of surgery. Additionally, as reported by Fransen BL et al., the use of the patella-in-place balancer in the surgical procedure contributed to prolonging the duration of the surgery. Since only two studies reported such significance, the effect of ERAS protocols on operation time is inconclusive.

Despite our primary interest in the current review, other clinical outcomes, including readmission rates, intraoperative blood losses, and mortality rates, were also reported. Those secondary outcomes are important indicators of a patient's reactivity following a surgical procedure.

Our findings showed that patients undergoing THA or TKA through an ERAS protocol experienced comparable readmission rates to those who received traditional care. Previous literature revealed similar outcomes regarding readmission rates among the two groups [29-31]. In contrast, readmission rates were reduced among the ERAS groups in comparison to control groups, as evidenced by an analysis conducted by Heymans MJ et al. for readmission, which presented a statistically significant difference in the enhanced recovery pathway in favor of knee arthroplasties (P=0.01) [32]. The author suggests that the significant effects of ERAS on readmission rates could be due to a clinical bias during data collection, which was possible because, as reported in the latter review, the data from large databases were used, and reporting bias is an issue in primary studies because of the selective outcomes reporting.

However, in our review, the ERAS groups significantly reduced intraoperative blood loss compared to traditional care in three of our included studies [19,21,22]. This could be attributed to the fact that blood management and intraoperative ERAS interventions are important components in controlling bleeding. For instance, tranexamic acid was commonly used by Azam MQ et al. (2022), Wei B et al. (2021), Ripolles-Melchor J et al. (2020), Jiang HH et al. (2019), and Tan NL et al. (2018) to reduce bleeding, which demonstrates its importance in intraoperative care and reducing blood loss. A previous analysis provided clinical evidence about the effect of the ERAS protocol on reducing blood losses among THA patients [32].

In addition, our findings showed that only two studies reported no occurrence of mortality following ERAS interventions [18,22]. However, other studies did not report data regarding mortality rates.

Limitations

Including studies from different geographic locations is essential to confirm the efficacy of ERAS protocols. However, more evidence to ensure the generalizability of this review has not been without limitations. Sources of bias were found in some studies. For instance, the statistical significance difference could be biased due to the small difference between the comparable groups in one of the included studies. Furthermore, the small sample size might have introduced some bias in one study. In addition, few studies were included in this review, and the heterogeneity limited further assessments of more clinical outcomes. However, identifying these limitations may help direct future studies to validate the impact of ERAS protocols on patient clinical outcomes, such as pain scores and functional recovery, using standardized measurements and protocols. Despite this limitation, the current review evaluation represents appropriate and up-to-date evidence of the ERAS effect on arthroplasty.

## Conclusions

This systematic review assesses the effect of ERAS protocols on THA and TKA patients' clinical outcomes. ERAS protocols significantly decreased the length of hospitalization. The effect on operation time was inconclusive, and the impact on readmission was comparable between the intervention and control groups. Despite this, the heterogeneity of the studies limited further assessments of more clinical outcomes. However, identifying these limitations could direct future studies to assess the efficacy of ERAS protocols on patient clinical outcomes using standardized approaches. Further research is needed to assess and analyze the definitive impact of each component of the ERAS protocol on TJA.
